# Intussusception and Other Adverse Event Surveillance after Pilot Introduction of Rotavirus Vaccine in Nam Dinh and Thua Thien Hue Provinces—Vietnam, 2017–2021

**DOI:** 10.3390/vaccines12020170

**Published:** 2024-02-07

**Authors:** Ly Khanh Thi Le, Thao Phuong Thi Pham, Le Thi Phuong Mai, Quyet Tu Nguyen, Mai Phuong Ngoc Tran, Thien Huu Ho, Hung Hoang Pham, Sanh Van Le, Ha Ngoc Hoang, Anh Tuan Lai, Nguyen Thuy Huong, Hien Dang Nguyen, Dang Duc Anh, Makiko Iijima, Umesh D. Parashar, Nguyen Van Trang, Jacqueline E. Tate

**Affiliations:** 1 National Institute of Hygiene and Epidemiology, Hanoi 100000, Vietnam; klylebio@gmail.com (L.K.T.L.); dda@nihe.org.vn (D.D.A.); 2 Center for Research and Production of Vaccines and Biologicals, Hanoi 100000, Vietnam; thaobiotech@gmail.com (T.P.T.P.); thuyhuong@polyvac.com.vn (N.T.H.);; 3 Central Hue Hospital, Thua Thien Hue 530000, Vietnam; thientrangduc@hotmail.com (T.H.H.);; 4 Hue Center for Disease Control, Thua Thien Hue 530000, Vietnam; 5 Nam Dinh General Hospital, Nam Dinh 420000, Vietnam; 6 Nam Dinh Center for Disease Control, Nam Dinh 420000, Vietnam; 7 World Health Organization, Vietnam Office, Hanoi 100000, Vietnam; iijimam@who.int; 8 United States Centers for Disease Control and Prevention, Atlanta, GA 30333, USA

**Keywords:** rotavirus, intussusception, Rotavin-M1 vaccine, safety, adverse events following immunization (AEFI)

## Abstract

Rotavin-M1 (POLYVAC) was licensed in Vietnam in 2012. The association of Rotavin-M1 with intussusception, a rare adverse event associated with rotavirus vaccines, and with adverse events following immunization (AEFI) have not been evaluated and monitored under conditions of routine use. From February 2017 to May 2021, we conducted a pilot introduction of Rotavin-M1 into the routine vaccination program in two provinces. Surveillance for intussusception was conducted at six sentinel hospitals. AEFI reports at 30 min and 7 days after vaccination were recorded. Among 443 children <12 months of age admitted for intussusception, most (92.3%) were children ≥ 6 months. Of the 388 children who were age-eligible to receive Rotavin-M1, 116 (29.9%) had received ≥1 dose. No intussusception cases occurred in the 1–21 days after dose 1 and one case occurred on day 21 after dose 2. Among the 45,367 children who received ≥1 dose of Rotavin-M1, 9.5% of children reported at least one AEFI after dose 1 and 7.3% after dose 2. Significantly higher AEFI rates occurred among children given Rotavin-M1 with pentavalent vaccines (Quinvaxem^®^, ComBE Five^®^) compared to Rotavin-M1 without pentavalent vaccines. There was no association between intussusception and Rotavin-M1. The vaccine was generally safe when administered alone and when co-administered with other vaccines.

## 1. Introduction

Rotavirus (RV) is the most common cause of several diarrhea in young children. However, the increasing use of rotavirus vaccines globally has contributed to a reduction in the burden of rotavirus disease [1,2,3]. In some studies, rotavirus vaccines have been reported to slightly increase the risk of intussusception [4,5,6,7,8,9]. Intussusception is a type of acute intestinal obstruction. When the small intestine becomes invaginated, the blood supply to the small intestine is lost, resulting in intestinal ischemia that can lead to perforation if not treated in a timely manner. Some intussusceptions resolve on their own, but most require hospitalization with treatment by enema or surgery. Mortality rates are highly dependent on timely access to appropriate treatment and range from less than 1% in developed countries to up to 10% in low income areas [10]. The incidence of intussusception peaks between 4 and 10 months of age and varies substantially by region [11]. A study from Ho Chi Minh City from 2009 to 2011 showed a relatively high baseline intussusception rate of 287/100,000 among children aged <1 year, which is higher than the intussusception rates in most other countries globally (global average, 74/100,000) [11,12,13,14].

Since 1999 when the first commercially available rotavirus vaccine, RotaShield^®^ (Wyeth-Ayerst, Philadelphia, Pennsylvania, USA), was linked with cluster of intussusception cases among vaccinees, subsequent rotavirus vaccines have undergone post-marketing surveillance for intussusception [15,16]. Post-marketing surveillance for both Rotarix^TM^ (GSK, Rixensart, Belgium) and RotaTeq^®^ (Merck & Co., Whitehouse Station, NJ, USA) vaccines showed an increased risk of intussusception in high- and middle-income countries. The risk of intussusception after the administration of Rotarix^TM^ was estimated to be 1.1–7.0 excess cases of intussusception per 100,000 infants vaccinated; RotaTeq^®^ was associated with approximately 1.5–7.3 excess cases per 100,000 recipients [5,6,7,8,9,17,18]. No increased risk of intussusception has been seen with Rotarix^TM^, RotaTeq^®^, or Rotavac^®^ (Bharat Biotech, India) in low-income countries [19,20,21,22].

Since 2012, a Vietnam-made rotavirus vaccine, Rotavin-M1 (POLYVAC, Vietnam), has been licensed and is available on the private market. The country plans to use this vaccine in the national expanded immunization program (EPI) over the coming years. In preparation for the nationwide introduction of this vaccine, a pilot vaccine introduction was conducted among approximately 45,000 children in 6 districts in 2 provinces (Nam Dinh and Thua Thien Hue (TT Hue)) from 2017 through 2021. During the pilot vaccine introduction, vaccine effectiveness was reported as 57% against rotavirus disease of all severities and 63% for severe rotavirus diarrhea [23]. The objective of the current analysis is to describe adverse events, including severe adverse events such as intussusception, that might be associated with Rotavin-M1 and which occurred during the pilot introduction.

## 2. Materials and Methods

### 2.1. Pilot Vaccine Introduction in Nam Dinh and TT Hue

Rotavin-M1 vaccine (G1P [8]) was pilot introduced into the expanded immunization program in some districts of Nam Dinh and TT Hue provinces from December 2017 to May 2021. Two doses of Rotavin-M1 vaccine were administered at 2 and 3 months of age alongside other routine infant immunizations. Information regarding rotavirus vaccine administration was recorded in community health center vaccination logbooks and the national electronic vaccination registry. Children who received at least one dose of Rotavin-M1 as part of the pilot introduction and satisfied the following inclusion criteria were included in the AEFI analysis: (1) Lived in one of the six participating districts; (2) received the first dose of Rotavin-M1 between 56–104 days of age; and (3) received the second dose of Rotavin-M1 before 6 months of age, if administered. Rotavin-M1 was available in parallel on the private market for children from 6 weeks of age.

### 2.2. Intussusception Surveillance

We conducted prospective intussusception surveillance among children less than 12 months of age from December 2017 to May 2021 in Nam Dinh and TT Hue provinces using six sentinel hospitals in these provinces. Duplicate cases were identified and removed, including transferred cases between hospitals in the network. These hospitals capture almost all of the intussusception cases occurring in the region. Children under 12 months of age admitted to one of these hospitals who met the Brighton Collaboration criteria for level 1 for diagnosing intussusception were included [24]. Medical staff completed a questionnaire on demographics, diagnostic and treatment methods, outcomes, child feeding practice, household information and vaccination history. The vaccination history for each child was obtained from either the immunization record books at commune health centers, the electronic immunization database, or the child’s personal vaccine card. 

### 2.3. Following up Intussusception Cases after Hospital Release

Beginning in November 2019, medical staff at the provincial CDC called the family after hospital release when the child turned 8 months of age to check the health status of the child. They verified demographic information and rotavirus vaccination status, documented the well-being after hospital discharge, and recorded whether any recurrent episodes of intussusception had occurred.

### 2.4. Adverse Events following Immunization (AEFI) Monitoring

Demographic characteristics (age, gender), vaccination information (date of administration, vaccination sites), and AEFIs after 30 min and after 7 days following the first and the second doses of Rotavin-M1 and co-administered vaccines (pentavalent/tetravalent vaccines and OPV) were collected and analyzed. Data were collected on standardized AEFI reporting forms completed by medical staff of each commune health center.

### 2.5. Statistical Analysis

Data were entered and cleaned using Epi Info software 7.2.5.0 (US-CDC, Atlanta, GA, USA) and analyzed by SPSS 22.0 software (IBM, New York, NY, USA). 

Intussusception surveillance data were analyzed descriptively for all enrolled children. To assess the association between intussusception and Rotavin-M1 administration, the analysis was restricted to children age-eligible to receive Rotavin-M1, which was defined as born on or after 8 September 2017 and enrolled on or after 25 December 2017. The risk window of interest was the 1 to 21 days after each dose of Rotavin-M1 vaccine.

For the AEFI data, chi-squared tests were used to compare two proportions of independent populations, and logistic binary regression models were used to estimate odds ratios and associated 95% confidence intervals to identify the factors influencing the occurrence of AEFIs of Rotavin-M1 in Vietnamese children.

### 2.6. Ethics Issues

The study was approved by the Ethics Committee of the National Institute of Hygiene and Epidemiology (IRB number—VN01057—19/2016) on 15 July 2016. Parents or legal representatives of participants signed the consent form to receive Rotavin-M1.

## 3. Results

### 3.1. Rotavin-M1 Pilot Introduction

During the 3 years of the pilot vaccine introduction (December 2017–May 2021), 45,367 children received at least one dose of Rotavin-M1; 88,214 doses of Rotavin-M1 were administered. In Nam Dinh province, 34,585 children received at least 1 dose and 32,863 children received two doses. In TT Hue province, number of children receiving at least one dose was 10,782 and 9984 received two doses. 

### 3.2. Characteristics of Intussusception Cases

From December 2017 to May 2021, a total of 443 hospitalizations (249 in Nam Dinh province and 194 in TT Hue province) for intussusception were reported among children <12 months of age who were enrolled at the six surveillance hospitals (Table 1). Intussusception cases occurred more frequently in boys (62.4–63.1%) than in girls (Table 1). Very few children younger than 3 months of age experienced an intussusception episode (0.2%) and only 7.5% of cases occurred in children 3–5 months of age. The majority of intussusception cases (92.3%) occurred in children 6–12 months of age (Table 1). Most (93.0%) cases were admitted within two days of symptom onset. The majority (97.1%) of cases were treated by air enema, while surgery accounted for 1.3% (6/443) of all cases. Surgery for all six cases was performed in Central Hue hospital; all these cases were aged 6–12 months, had a time from symptom onset to hospital admission of 2–4 days, and 66.6% (4/6) required intestinal resection. Only seven cases (1.6%) were not treated successfully at a district hospital and were transferred to higher level hospitals. Most (98.4%) children treated for intussusception had a short hospital stay (1–7 days). For all children, the mean hospital stay was 2.1 days in Nam Dinh and 3.4 days in TT Hue province (Table 1). Longer stay of 15 days or more often occurred when other diseases required transfer to another department for treatment (pneumonia, diarrhea, etc.). Of the six children who required surgery, each was hospitalized for more than eight days. Most children (98.4%) were successfully treated for intussusception at the original admission hospital and discharged home. From November 2019 to May 2021, provincial CDC staff attempted to contact children that were discharged before 8 months of age. Of the 144 children successfully contacted, nine (6.3%) children had a recurrent episode of intussusception.

### 3.3. Association between Rotavirus Vaccination and Intussusception

Of the 443 children enrolled with intussusception, the number of children that were age eligible to receive Rotavin-M1 (who were born on or after 8 September 2017 and enrolled on or after 25 December 2017) was 388 (87.6%); this included 222 children from Nam Dinh and 166 children from TT Hue. In Nam Dinh province, 80 patients (36.0%) received the first dose of Rotavin-M1 vaccine and 69 (31.1%) received the second dose of Rotavin-M1. In TT Hue province, 36 (21.7%) patients received the first dose of Rotavin-M1 and 29 (17.5%) patients received the second dose. A total of 48 (12.4%) children age-eligible for vaccine were excluded from further analysis because they had received either the RotaTeq^®^ or Rotarix^TM^ vaccines.

**Figure 1:**
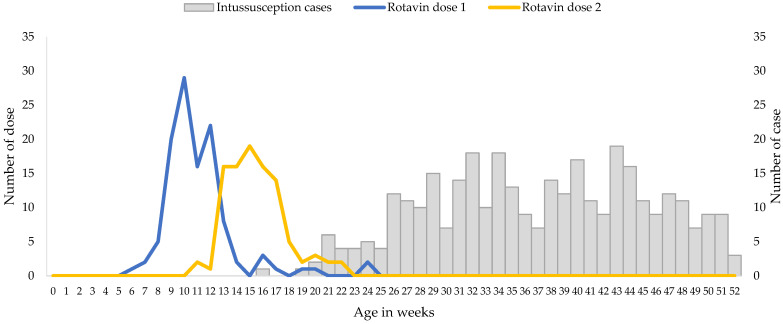
Ages at Immunization and at Onset of Intussusception, December 2017 through May 2021. Gray bars indicate the numbers of intussusception cases according to age at symptom onset, and the blue and yellow lines indicate the numbers of doses of Rotavin-M1 administered according to age at immunization.

The median age of the infants included in the analysis was 37.5 weeks, with very few cases of intussusception detected in children less than 20 weeks of age (Figure 1).

Rotavin-M1 vaccination coverage was low but the receipt of the vaccine doses were timely, i.e., all received two doses before six months of age as per manufacturer recommendation, and approximately 90% of children received the first dose by 14 weeks (Figure 1). A total of 65.9% of patients with intussusception that were included in the analysis were unvaccinated, 5.3% had received only 1 dose of Rotavin-M1, and 28.8% received 2 doses of Rotavin-M1. Median age at dose 1 was 10 weeks (interquartile range 9.5–12 weeks) and the median age at dose 2 was 15 weeks (interquartile range 14–17 weeks).

**Figure 2:**
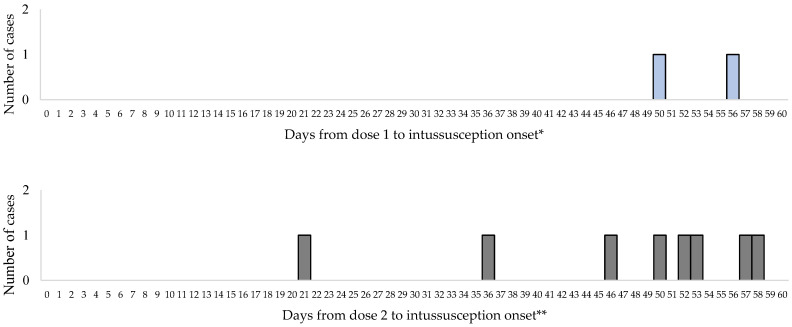
Interval between Rotavin-M1 administration and intussusception onset by dose. * 114 additional cases occurred at greater than 60 days post Rotavin-M1 dose 1 and ** 91 additional cases occurred at greater than 60 days post Rotavin-M1 dose 2 were not included.

No cases of intussusception occurred within 1 to 7 days or 8 to 21 days following the first dose of Rotavin-M1 vaccine. The first intussusception case after the first dose of Rotavin-M1 occurred on day 50. No cases of intussusception occurred within 1 to 7 days after dose 2 of Rotavin-M1 and one case occurred on day 21 following dose 2 (Figure 2).

### 3.4. Adverse Events following Immunization (AEFI)

From December 2017 to May 2021, adverse events records were obtained for 45,367 children of the more than 50,000 subjects who had received at least one dose of Rotavin-M1. The prevalence of children who experienced at least one AEFI within 7 days after the first dose of Rotavin-M1 and other vaccines was 9.5% (*n* = 4294). The prevalence of AEFIs after the second dose was 7.3% (*n* = 3125). AEFIs after the first dose were more frequent than after the second dose at all sites (Table 2). Males and females showed similar AEFI frequency (Table 2). The rate of reporting AEFIs after the first (11.3%) and second (8.7%) dose in Nam Dinh was over three times higher than in TT Hue, with 3.5% and 2.6% after the first doses and second doses, respectively. Rates of AEFIs in the second year (December 2018 to November 2019) and third year (December 2019 to December 2020) of the pilot project were 2–3 times higher compared with the first year of introduction of Rotavin-M1 (December 2017 to November 2018) (Table 2).

The frequency of AEFIs in children who received Rotavin-M1 and OPV (without pentavalent/tetravalent vaccine) on the same day or within 7 days of one another was not significantly different compared with those received Rotavin-M1 without OPV co-administered within 7 days (approximately 3% in both groups). In contrast, all children who were administered Rotavin-M1 and pentavalent/tetravalent vaccine on the same day or within 7 days of one another (with or without OPV) showed a higher rate of AEFIs than among children administered Rotavin-M1 and pentavalent/tetravalent vaccines separately, ranging 9.0% to 11.9%. The rate of AEFIs in children co-administered with Rotavin-M1 and pentavalent/tetravalent vaccine within 7 days increased markedly compared with using only Rotavin-M1 or Rotavin-M1 and OPV/IPV.

The main symptom reported within 30 min of rotavirus vaccine co-administered with pentavalent vaccine and OPV was vomiting (0.9% and 0.6% after the 1st and 2nd dose, respectively). During the 7 days after vaccination, pyrexia occurred in 8.4% and 6.5% of children after 1st and 2nd dose, respectively. Other symptoms such as diarrhea, vomiting, fuzziness, pain, and/or swelling occurred but at very low frequencies during the 7 days post vaccination (Table 3).

## 4. Discussion

In this study, we found that no cases of intussusception occurred in the 1 to 21 days after dose 1 of Rotavin-M1 and only one case occurred in the 1 to 21 days after dose 2 of this vaccine. Thus, no intussusception cases were seen in the 1–7 day risk window, with nearly 45,000 first doses administered. While the lack of cases precluded a formal risk assessment using the self-controlled case-series design, it suggests that the risk, if any, is likely to be quite low. Although Vietnam is among the countries with the highest rates of intussusception globally, no cases of intussusception occurred in children <3 months of age and only 8% of cases occurred in children 3 to 5 months of age [24]. Thus, intussusception cases rarely occurred in young infants in Vietnam when the doses of Rotavin-M1 were administered. 

Our first-ever-in-Vietnam finding that there is no evidence of an association between intussusception and Rotavin-M1 vaccination is in line with other studies in low and low-middle–income countries and in sub-Saharan African countries that use Rotarix^TM^ and RotaTeq^®^ [20,21,22,25]. Of note, Rotavin-M1 and Rotarix^TM^ are both based on G1P [8] rotavirus vaccine strains that were derived from human infants [19,26,27]. These findings are in contrast with those from high and upper-middle income countries where there is an association between rotavirus vaccination and intussusception during the first week after the first rotavirus vaccine dose [5,6,7,8,9,28,29]. There are several possible explanations. First, in a comparison between Rotavin-M1 and Rotarix^TM^, shedding of Rotavin-M1 vaccine strain was delayed and in a smaller proportion of children compared to Rotarix^TM^, suggesting limited replication of the former in our children [27,30]. Second, a study in South African infants found that co-administration of OPV and rotavirus vaccine led to lower immunogenicity of the rotavirus vaccine first dose [31]. This finding suggests that children who receive OPV and the oral rotavirus vaccine at the same time may be less likely to mount an immune response that could trigger intussusception than children who have inactivated polio vaccine co-administered with oral rotavirus vaccine. In our pilot vaccine introduction, Rotavin-M1 was administered at the same time as OPV and DPT-Hib-HepB (Quinvaxem^®^ (Berna biotech, Korea) or vaccine from Serum Institute of India (SII)) in 95% of children and on a designated day of the month at commune health centers of the six districts of the study. Finally, other factors such as breast feeding, microbiome, or the maternal antibody might also lead to the difference in intussusception rates after rotavirus vaccination [32,33,34].

A very low rate of surgery was observed in Vietnam (1.3% overall) and no mortality was found during the 3-year study period. This finding is in stark contrast to other settings where no risk of intussusception has been observed following rotavirus vaccination but almost all children were treated by surgery and the mortality rate was over 10% [21,29,35]. One possible reason for the low mortality rate in Vietnam is that the widespread use of ultrasound imaging might lead to early diagnosis of diseases and early presentation of the child to medical facilities for treatment (often within 1–2 days of symptom manifestation).

We also report results from the first post-marketing surveillance of Rotavin-M1 vaccine when used with other vaccines in the routine immunization program. The prevalence of AEFIs after the first dose of vaccine (9.5%) was higher than after the second dose (7.3%) in all study sites. This trend was similar to that observed in previous clinical studies of Rotavin where the expected AEs after the first dose and the second dose, respectively, were 27.8% and 17.0% [36]. The risk of AEFIs increased more than 4–6 times when Rotavin-M1 and pentavalent/tetravalent vaccine were co-administered simultaneously. In Vietnam, pentavalent vaccines used in the routine program such as: (1) Quinvaxem^®^ (Berna biotech, Korea), (2) Diphtheria, Tetanus, Pertussis, Hepatitis B, and Haemophilus influenzae type b Conjugate Vaccine Adsorbed (SII, India), and (3) ComBE Five^®^ (Biological E, India) with whole-cell pertussis component. These vaccines may have resulted in a higher rate of fever and local reactions (swelling, heat, redness, pain at the injection site) after co-administration (13.3–19.1%), while the rate of AEFIs after either dose of Rotavin-M1 was 2.4–3.7%. A higher rate of fever after co-administration of the rotavirus and other childhood vaccines was also reported by the Oxford Royal College of General Practitioners Research and Surveillance Centre (1.62% and 0.5%, respectively) [37]. The other symptoms observed in this study were fever, diarrhea, and vomiting, which were reported with low frequency (<1%). These symptoms were found at lower frequencies compared to those reported in clinical studies due to the passive AEFI reporting method. In particular, a study in Vietnam about the safety of Quinvaxem^®^ in infants at 2 to 4 months of age showed that overall, 70.7% of children experienced at least one solicited adverse event, of which 33.0% experienced at least one solicited local adverse event and 37.7% experienced at least one solicited systemic adverse event. The most common local reactions were swelling and pain at the infection site, at a frequency of 5.6% (ranging 0% to 14.5% between doses) and 4.1% (ranging 0% to 18.3%) of the combined vaccine doses, respectively. Fever (18.4%, range 13.8% to 26.0%) and irritability (7.9%, range 1.5% to 16.6%) were the most common solicited systemic adverse events, whereas low frequency of diarrhea (3.0%, range 2.3% to 3.8%), loss of appetite (2.6%, range 0% to 7.6%), vomiting (1.0%, range 0% to 3%), and persistent crying and drowsiness/sleepiness (both 0.5%, range 0% to 1.5%) were reported. Most systemic adverse events were mild and very few severe events were reported [38].

Our study has limitations. First, an insufficient number of children with intussusception were enrolled during the risk windows following vaccination to conduct a self-controlled case-series analysis. This may have been due to the global COVID-19 pandemic began in 2020 which may have affected surveillance and resulted in an under detection of intussusception cases. However, it is unlikely that the under detection would have been age related and given the age distribution of intussusception cases observed in this analysis with very low numbers of intussusception cases in young infants, naturally occurring intussusception cases are likely rare at the time of rotavirus vaccine administration in Vietnam. Second, reporting of AEFIs was variable by province and over time, which makes calculating the risk of AEFIs challenging. Better reporting and capture of AEFIs over time may explain the increase in AEFIs by year and the higher rates of AEFIs in Nam Dinh compared with TT Hue. Finally, an increased risk of AEFIs was associated with co-administration of Rotavin-M1 and pentavalent/tetravalent vaccine but this may be due to the pentavalent/tetravalent vaccine and not the rotavirus vaccine. Finally, the global COVID-19 pandemic began in the third year post-vaccine introduction and might seem to affect care-seeking behavior during this period. However, our study showed that the rate of AEFI was higher in this period compared to pre-COVID-19 periods which may reflect the heightened awareness of both medical staff and children parents/guardians for symptom reporting after vaccination.

## 5. Conclusions

In this study, after 3.5 years of intussusception surveillance, there was no evidence to suggest an association between intussusception and Rotavin-M1 vaccination. The prevalence of AEFI was significantly higher in children vaccinated with Rotavin-M1 and pentavalent vaccine than in children who received Rotavin-M1 vaccine alone.

## Figures and Tables

**Table 1:** Characteristics of intussusception pediatric patients under 1 year old admitted to hospitals in Nam Dinh and TT Hue provinces from December 2017–May 2021.

	Nam Dinh *(n* = 249)	TT Hue (*n* = 194)	Total (*N* = 443)	*p*-Value *
Surveillance year				
December 2017–November 2018, n (%)	73 (29.3)	55 (28.4)	128 (28.9)	
December 2018–November 2019, n (%)	81 (32.5)	61 (31.4)	142 (32.1)	0.91
December 2019–May 2021, n (%)	95 (38.2)	78 (40.2)	173 (39.0)	
Age (months)				
<3 months, n (%)	1 (0.4)	0 (0.0)	1 (0.2)	
3–5 months, n (%)	16 (6.4)	17 (8.8)	33 (7.5)	0.38
6–8 months, n (%)	124 (49.8)	84 (43.3)	208 (47.0)	
9–12 months, n (%)	108 (43.4)	93 (47.9)	201 (45.3)	
Gender				
Male, n (%)	157 (63.1)	121 (62.4)	278 (62.8)	0.88
Symptoms onset—hospital admission (days)				
1–2, n (%)	237 (95.2)	175 (90.2)	412 (93.0)	0.01
3–7, n (%)	9 (3.6)	19 (9.8)	28 (6.3)	
UNKNOWN, n (%)	3 (1.2) ^a^	0 (0.0)	3 (0.7)	
Range	1–6	1–6		
Mean (SD)	1.4 (0.6)	1.5 (0.8)		
Treatment				
Air enema, n (%)	244 (98.0)	186 (95.9)	430 (97.1)	
Surgery, n (%)	0 (0.0)	6 (3.1)	6 (1.3)	
Hospital transfer, n (%)	5 (2.0)	2 (1.0)	7 (1.6)	0.02
Hospital duration (days)				
1–2, n (%)	211 (84.7)	62 (32.0)	273 (61.6)	
3–7, n (%)	37 (14.9)	126 (65.0)	163 (36.8)	
8–14, n (%)	0 (0.0)	3 (1.5) ^c^	3 (0.7)	
>15, n (%)	0 (0.0)	3 (1.5) ^c^	3 (0.7)	0.00
UNKNOWN, n (%)	1 (0.4) ^b^	0 (0.0)	1 (0.2)	
Range	1–4	1–23		
Mean (SD)	2.1 (0.6)	3.4 (2.4)		

^a^ cases missing date of symptoms onset. ^b^ case with missing hospital discharge date. ^c^ case of surgery. * *p*-value compares the distribution of the characteristics of intussusception pediatric patients in Nam Dinh and TT Hue.

**Table 2:** Relationship between adverse events after immunization with related factors.

		Adverse Events after the First Dose	Adverse Events after the Second Dose	*p*-Value *
		*n/N* (%)	*n/N* (%)	
Gender	Male	2240/23,746 (9.4)	1625/22,451(7.2)	0.88
	Female	2054/21,621 (9.5)	1500/20,396 (7.4)	
Location	TT Hue	380/10,782 (3.5)	255/9984 (2.6)	0.29
	Nam Dinh	3914/34,585 (11.3)	2870/32,863 (8.7)	
Year	December 2017–November 2018	784/16,027 (4.9)	580/15,208 (3.8)	0.81
	December 2018–November 2019	1482/14,975 (9.9)	1093/14,164 (7.7)	
	December 2019–May 2021	2028/14,365 (14.1)	1452/13,475 (10.8)	
Only Rotavin-M1		4/107 (3.7)	6/247 (2.4)	0.49
Combination (within one week)	Rotavin-M1 and OPV/IPV (without pentavalent vaccine)	17/546 (3.1)	13/914 (1.4)	0.18
	Rotavin-M1 and pentavalent vaccine (without OPV/IPV)	18/94 (19.1)	5/54 (9.3)	
	Rotavin-M1 and pentavalent vaccine and OPV	4033/33,981 (11.9)	2742/30,482 (9.0)	
Combination (more than one week)	Rotavin-M1 and OPV/IPV (with pentavalent vaccine)	177/9281 (1.9)	290/9472 (3.1)	0.00
	Rotavin-M1 and pentavalent vaccine (with OPV/IPV)	4/30 (13.3)	12/61 (19.7)	
	Rotavin-M1 and pentavalent vaccine and OPV	40/1289 (3.1)	24/1165 (2.1)	
Total		4294/45,367 (9.5)	3125/42,847 (7.3)	

* *p*-value compares the distribution of the characteristics of adverse events after immunization.

**Table 3:** Number of children who experienced at least of one common AEFIs of Rotavin-M1 and other vaccines by symptoms, monitoring duration by study province, from December 2017 to May 2021.

Location	Dose	*N*	AEFI Cases by Time of Monitoring (%)					
			30 min		7 days			
			Vomiting	Pyrexia	Vomiting	Diarrhea	Pyrexia	Others *
TT Hue	1st	10,786	48 (0.4%)	0 (0.0%)	2 (0.0%)	4 (0.0%)	330 (3.1%)	4 (0.0%)
	2nd	9984	24 (0.2%)	0 (0.0%)	0 (0.0%)	3 (0.0%)	223 (2.2%)	9 (0.1%)
Nam Dinh	1st	34,586	371 (1.1%)	15 (0.0%)	13 (0.0%)	27 (0.1%)	3475 (10.0%)	13 (0.6%)
	2nd	32,865	234 (0.7%)	21 (0.1%)	7 (0.0%)	22 (0.1%)	2471 (7.8%)	171 (0.5%)
Total	1st	45,372	419 (0.9%)	15 (0.0%)	15 (0.0%)	31 (0.1%)	3805 (8.4%)	17 (0.4%)
	2nd	42,849	258 (0.6%)	21 (0.0%)	7 (0.0%)	25 (0.1%)	2694 (6.5%)	180 (0.4%)

* Other AES were observed rarely such as fuzziness, crying, swelling, pain.

## Data Availability

Data sharing inquiries should be directed to the authors.

## References

[1] Troeger C., Colombara D.V., Rao P.C., Khalil I.A., Brown A., Brewer T.G., Guerrant R.L., Houpt E.R., Kotloff K.L., Misra K. (2018). Global disability-adjusted life-year estimates of long-term health burden and undernutrition attributable to diarrhoeal diseases in children younger than 5 years. Lancet Glob. Health.

[2] Operario D.J., Platts-Mills J.A., Nadan S., Page N., Seheri M., Mphahlele J., Praharaj I., Kang G., Araujo I.T., Leite J.P.G. (2017). Etiology of Severe Acute Watery Diarrhea in Children in the Global Rotavirus Surveillance Network Using Quantitative Polymerase Chain Reaction. J. Infect. Dis..

[3] GBDDD C. (2018). Estimates of the global, regional, and national morbidity, mortality, and aetiologies of diarrhoea in 195 countries: A systematic analysis for the Global Burden of Disease Study 2016. Lancet Infect. Dis..

[4] Abramson J.S., Baker C.J., Fisher M.C., Gerber M.A., Meissner H.C., Murray D.L., Overturf G.D., Prober C.G., Rennels M.B., Saari T.N. (1999). Possible association of intussusception with rotavirus vaccination. American Academy of Pediatrics. Committee on Infectious Diseases. Pediatrics.

[5] Weintraub E.S., Baggs J., Duffy J., Vellozzi C., Belongia E.A., Irving S., Klein N.P., Glanz J.M., Jacobsen S.J., Naleway A. (2014). Risk of intussusception after monovalent rotavirus vaccination. N. Engl. J. Med..

[6] Yih W.K., Lieu T.A., Kulldorff M., Martin D., McMahill-Walraven C.N., Platt R., Selvam N., Selvan M., Lee G.M., Nguyen M. (2014). Intussusception risk after rotavirus vaccination in U.S. infants. N. Engl. J. Med..

[7] Stowe J., Andrews N., Ladhani S., Miller E. (2016). The risk of intussusception following monovalent rotavirus vaccination in England: A self-controlled case-series evaluation. Vaccine.

[8] Carlin J.B., Macartney K.K., Lee K.J., Quinn H.E., Buttery J., Lopert R., Bines J., McIntyre P.B. (2013). Intussusception risk and disease prevention associated with rotavirus vaccines in Australia’s National Immunization Program. Clin. Infect. Dis. Off. Publ. Infect. Dis. Soc. Am..

[9] Yung C.F., Chan S.P., Soh S., Tan A., Thoon K.C. (2015). Intussusception and Monovalent Rotavirus Vaccination in Singapore: Self-Controlled Case Series and Risk-Benefit Study. J. Pediatr..

[10] Jiang J., Jiang B., Parashar U., Nguyen T., Bines J., Patel M.M. (2013). Childhood intussusception: A literature review. PLoS ONE.

[11] World Health Organization (2002). Acute Intussusception in Infants and Children Incidence, Clinical Presentation and Management: A Global Perspective.

[12] Jo D.S., Nyambat B., Kim J.S., Jang Y.T., Ng T.L., Bock H.L., Kilgore P.E. (2009). Population-based incidence and burden of childhood intussusception in Jeonbuk Province, South Korea. Int. J. Infect. Dis. IJID Off. Publ. Int. Soc. Infect. Dis..

[13] Bines J.E., Liem N.T., Justice F.A., Son T.N., Kirkwood C.D., de Campo M., Barnett P., Bishop R.F., Robins-Browne R., Carlin J.B. (2006). Risk factors for intussusception in infants in Vietnam and Australia: Adenovirus implicated, but not rotavirus. J. Pediatr..

[14] Dong A.T., Mong H.T., Van B.N. (1999). Acute intestinal invagination: Pneumatic reduction (experience with 2033 cases). Arch. Pediatr. Organe Off. Soc. Fr. Pediatr..

[15] Schwartz J.L. (2012). The first rotavirus vaccine and the politics of acceptable risk. Milbank Q..

[16] Murphy T.V., Gargiullo P.M., Massoudi M.S., Nelson D.B., Jumaan A.O., Okoro C.A., Zanardi L.R., Setia S., Fair E., LeBaron C.W. (2001). Intussusception among infants given an oral rotavirus vaccine. N. Engl. J. Med..

[17] Patel M.M., López-Collada V.R., Bulhões M.M., De Oliveira L.H., Bautista Márquez A., Flannery B., Esparza-Aguilar M., Montenegro Renoiner E.I., Luna-Cruz M.E., Sato H.K. (2011). Intussusception risk and health benefits of rotavirus vaccination in Mexico and Brazil. N. Engl. J. Med..

[18] Buttery J.P., Danchin M.H., Lee K.J., Carlin J.B., McIntyre P.B., Elliott E.J., Booy R., Bines J.E. (2011). Intussusception following rotavirus vaccine administration: Post-marketing surveillance in the National Immunization Program in Australia. Vaccine.

[19] Bergman H., Henschke N., Hungerford D., Pitan F., Ndwandwe D., Cunliffe N., Soares-Weiser K. (2021). Vaccines for preventing rotavirus diarrhoea: Vaccines in use. Cochrane Database Syst. Rev..

[20] Tate J.E., Mwenda J.M., Keita A.M., Tapsoba T.W., Ngendahayo E., Kouamé B.D., Samateh A.L., Aliabadi N., Sissoko S., Traore Y. (2024). Evaluation of Intussusception Following Pentavalent Rotavirus Vaccine (RotaTeq) Administration in Five Countries in Africa. Clin. Infect. Dis. Off. Publ. Infect. Dis. Soc. Am..

[21] Burnett E., Riaz A., Anwari P., Myat T.W., Chavers T.P., Talat N., Safi N., Aung N.N.T., Cortese M.M., Sultana S. (2023). Intussusception risk following oral monovalent rotavirus vaccination in 3 Asian countries: A self-control case series evaluation. Vaccine.

[22] Reddy S.N., Nair N.P., Tate J.E., Thiyagarajan V., Giri S., Praharaj I., Mohan V.R., Babji S., Gupte M.D., Arora R. (2020). Intussusception after Rotavirus Vaccine Introduction in India. N. Engl. J. Med..

[23] Van Trang N., Tate J.E., Phuong Mai L.T., Vu T.D., Quyet N.T., Thi Le L.K., Thi Chu M.N., Ngoc Tran M.P., Thi Pham T.P., Nguyen H.T. (2023). Impact and effectiveness of Rotavin-M1 under conditions of routine use in two provinces in Vietnam, 2016–2021, an observational and case-control study. Lancet Reg. Health West. Pac..

[24] Bines J.E., Kohl K.S., Forster J., Zanardi L.R., Davis R.L., Hansen J., Murphy T.M., Music S., Niu M., Varricchio F. (2004). Acute intussusception in infants and children as an adverse event following immunization: Case definition and guidelines of data collection, analysis, and presentation. Vaccine.

[25] Groome M.J., Tate J.E., Arnold M., Chitnis M., Cox S., de Vos C., Kirsten M., le Grange S.M., Loveland J., Machaea S. (2020). Evaluation of Intussusception after Oral Monovalent Rotavirus Vaccination in South Africa. Clin. Infect. Dis. Off. Publ. Infect. Dis. Soc. Am..

[26] Le L.T., Nguyen T.V., Nguyen P.M., Huong N.T., Huong N.T., Huong N.T.M., Hanh T.B., Ha D.N., Anh D.D., Gentsch J.R. (2009). Development and characterization of candidate rotavirus vaccine strains derived from children with diarrhoea in Vietnam. Vaccine.

[27] Dang D.A., Nguyen V.T., Vu D.T., Nguyen T.H., Nguyen D.M., Yuhuan W., Baoming J., Nguyen D.H., Le T.L. (2012). A dose-escalation safety and immunogenicity study of a new live attenuated human rotavirus vaccine (Rotavin-M1) in Vietnamese children. Vaccine.

[28] Leino T., Ollgren J., Strömberg N., Elonsalo U. (2016). Evaluation of the Intussusception Risk after Pentavalent Rotavirus Vaccination in Finnish Infants. PLoS ONE.

[29] Fotso Kamdem A., Vidal C., Pazart L., Leroux F., Pugin A., Savet C., Sainte-Claire Deville G., Guillemot D., Massol J. (2019). A case-control study of risk factors for intussusception among infants in eastern France after the introduction of the rotavirus vaccine. Vaccine.

[30] Skansberg A., Sauer M., Tan M., Santosham M., Jennings M.C. (2021). Product review of the rotavirus vaccines ROTASIIL, ROTAVAC, and Rotavin-M1. Hum. Vaccines Immunother..

[31] Steele A.D., De Vos B., Tumbo J., Reynders J., Scholtz F., Bos P., de Beer M.C., Van der Merwe C.F., Delem A. (2010). Co-administration study in South African infants of a live-attenuated oral human rotavirus vaccine (RIX4414) and poliovirus vaccines. Vaccine.

[32] Patel M., Shane A.L., Parashar U.D., Jiang B., Gentsch J.R., Glass R.I. (2009). Oral rotavirus vaccines: How well will they work where they are needed most?. J. Infect. Dis..

[33] Velasquez D.E., Parashar U., Jiang B. (2018). Decreased performance of live attenuated, oral rotavirus vaccines in low-income settings: Causes and contributing factors. Expert Rev. Vaccines.

[34] Kirkpatrick B.D., Colgate E.R., Mychaleckyj J.C., Haque R., Dickson D.M., Carmolli M.P., Nayak U., Taniuchi M., Naylor C., Qadri F. (2015). The “Performance of Rotavirus and Oral Polio Vaccines in Developing Countries” (PROVIDE) study: Description of methods of an interventional study designed to explore complex biologic problems. Am. J. Trop. Med. Hyg..

[35] Quinn H.E., Wood N.J., Cannings K.L., Dey A., Wang H., Menzies R.I., Moberley S., Reid S., McIntyre P.B., Macartney K.K. (2014). Intussusception after monovalent human rotavirus vaccine in Australia: Severity and comparison of using healthcare database records versus case confirmation to assess risk. Pediatr. Infect. Dis. J..

[36] Thiem V.D., Anh D.D., Ha V.H., Hien N.D., Huong N.T., Nga N.T., Thang T.C., McNeal M.M., Meyer N., Pham H.L. (2021). Safety and immunogenicity of two formulations of rotavirus vaccine in Vietnamese infants. Vaccine.

[37] Bauwens J., de Lusignan S., Weldesselassie Y.G., Sherlock J., Künzli N., Bonhoeffer J. (2022). Safety of routine childhood vaccine coadministration versus separate vaccination. BMJ Glob. Health.

[38] Huu T.N., Phuong N.T., Toan N.T., Thang H.V. (2015). Immunogenicity and safety of Quinvaxem® (diphtheria, tetanus, whole-cell pertussis, hepatitis B and haemophilus influenzae Type B vaccine) given to vietnamese infants at 2 to 4 months of age. Southeast Asian J. Trop. Med. Public Health.

